# Differences in the organisation of early pregnancy units and the effect of senior clinician presence, volume of patients and weekend opening on emergency hospital admissions: Findings from the VESPA Study

**DOI:** 10.1371/journal.pone.0260534

**Published:** 2021-11-30

**Authors:** Maria Memtsa, Venetia Goodhart, Gareth Ambler, Peter Brocklehurst, Edna Keeney, Sergio A. Silverio, Zacharias Anastasiou, Jeff Round, Nazim Khan, Jennifer Hall, Geraldine Barrett, Ruth Bender-Atik, Judith Stephenson, Davor Jurkovic

**Affiliations:** 1 Elizabeth Garrett Anderson Institute for Women’s Health, University College London, London, United Kingdom; 2 Department of Statistical Science, University College London, London, United Kingdom; 3 Birmingham Clinical Trials Unit, Institute of Applied Health Research, University of Birmingham, Birmingham, United Kingdom; 4 Population Health Sciences, Bristol Medical School, University of Bristol, Bristol, United Kingdom; 5 Department of Women and Children’s Health, King’s College London, London, United Kingdom; 6 Institute of Health Economics, Edmonton, Canada; 7 Modelling and Analytical Systems Solutions (MASS) Ltd, Edinburgh, United Kingdom; 8 The Miscarriage Association, Wakefield, United Kingdom; Nantong University, CHINA

## Abstract

**Objective:**

To determine whether the participation of consultant gynaecologists in delivering early pregnancy care results in a lower rate of acute hospital admissions.

**Design:**

Prospective cohort study and emergency hospital care audit; data were collected as part of the national prospective mixed-methods VESPA study on the “**V**ariations in the organization of **E**PAUs in the UK and their effects on clinical, **S**ervice and **PA**tient-centred outcomes”.

**Setting:**

44 Early Pregnancy Assessment Units (EPAUs) across the UK randomly selected in balanced numbers from eight pre-defined mutually exclusive strata.

**Participants:**

6606 pregnant women (≥16 years old) with suspected first trimester pregnancy complications attending the participating EPAUs or Emergency Departments (ED) from December 2016 to July 2017.

**Exposures:**

Planned and actual senior clinician presence, unit size, and weekend opening.

**Main outcome measures:**

Unplanned admissions to hospital following any visit for investigations or treatment for first trimester complications as a proportion of women attending EPAUs.

**Results:**

205/6397 (3.2%; 95% CI 2.8–3.7) women were admitted following their EPAU attendance. The admission rate among 44 units ranged from 0% to 13.7% (median 2.8). Neither planned senior clinician presence (p = 0.874) nor unit volume (p = 0.247) were associated with lower admission rates from EPAU, whilst EPAU opening over the weekend resulted in lower admission rates (p = 0.027). 1445/5464 (26.4%; 95%CI 25.3 to 27.6) women were admitted from ED. There was little evidence of an association with planned senior clinician time (p = 0.280) or unit volume (p = 0.647). Keeping an EPAU open over the weekend for an additional hour was associated with 2.4% (95% CI 0.1% to 4.7%) lower odds of an emergency admission from ED.

**Conclusions:**

Involvement of senior clinicians in delivering early pregnancy care has no significant impact on emergency hospital admissions for early pregnancy complications. Weekend opening, however, may be an effective way of reducing emergency admissions from ED.

## Introduction

Current constraints on health expenditure have compelled providers to explore ways of delivering a greater proportion of care in outpatient settings whilst maintaining good clinical outcomes and high levels of patient satisfaction. In the UK, the care of women experiencing complications in the first trimester of pregnancy is mainly provided in Early Pregnancy Assessment Units (EPAUs). They are organisational structures which operate in most acute hospitals in the UK and an increasing number of other countries worldwide [1–4]. They were established following a publication by Bigrigg and Read [5] in 1991 who reported on cost savings and improved quality of early pregnancy care following the opening of an EPAU in their local hospital. EPAUs are currently operating in most acute hospital units in the UK aiming to provide women with comprehensive early pregnancy care, which includes clinical and ultrasound assessment, laboratory investigations, further management, as well as counselling and support, all in an out-patient setting.

Research that could potentially guide recommendations and policies on staffing and configurations of EPAUs has been scarce [6–9]. The National Institute for Health and Care Excellence (NICE) guideline on Ectopic Pregnancy and Miscarriage (CG 154) [10] included a review of evidence comparing EPAUs with other models of providing early pregnancy care for women presenting with first trimester pregnancy complications. The NICE guideline developing group, which includes a patient and public involvement panel, concluded that the configuration of services between different units varied considerably but, due to paucity of data, they were unable to make recommendations on the optimal structure of EPAUs that would balance clinical and service outcomes against cost-effectiveness. As a result, the group prioritised the need for future good quality research into the relative effectiveness of different EPAU configurations [10]. Senior clinician (consultant gynaecologist) involvement in the delivery of acute clinical care has been of interest recently [11], as there is emerging evidence of their positive impact on Emergency Departments (EDs) and Acute Medical Units, contributing to reduced mortality, fewer emergency hospital admissions and accelerated discharge from hospital [12–14].

In this national prospective cohort study, we aimed to determine whether higher participation of senior clinicians in the delivery of first trimester pregnancy care would result in better quality clinical care reflected in a lower risk of acute admissions to hospital for women presenting with first trimester complications.

## Methods

### Study setting

Data for this observational study were collected as part of the VESPA study, a UK-wide prospective mixed-methods study on the “**V**ariations in the organization of **E**PAUs in the UK and their effects on clinical, **S**ervice and **PA**tient-centred outcomes”. We report the data from two of the VESPA study strands: 1) the prospective cohort study of women attending EPAUs due to first trimester complications; and 2) the emergency hospital care audit of women admitted as an emergency with first trimester pregnancy complications.

Prior to unit recruitment, a national survey was distributed to all 212 EPAUs operating in UK NHS hospitals between 2015 and 2016. A total of 205 EPAUs which completed the survey reported on unit location, opening hours, access to services, and staffing configurations. These responses were used to categorize the units according to three factors: planned senior clinician presence (yes or no); weekend opening (yes or no); and the annual volume of patients, as reported by the corresponding clinicians (low volume <3000 appointments per year vs. high volume ≥3000 appointments annually). These three factors were selected as being important factors associated with clinical outcomes from the literature [6]. Units were divided into eight mutually exclusive strata based on these characteristics, and 5 or 6 EPAUs were randomly selected from each stratum to a total of 44 for inclusion in the VESPA study. This technique was adopted to ensure that units of all configurations were equally represented in the final sample. The unit characteristics were confirmed again immediately prior to commencing patient recruitment.

### Patient involvement

Patients and members of the public were involved at several stages of the study, including the design, management, and conduct of the study. We received input from patients who had experienced first trimester pregnancy complications in the design and timing of the study materials, and management oversight through membership of the study steering committee. A user-led organization (Miscarriage Association), through its national director, acted as a co-applicant and collaborator. We carefully assessed the burden and timings of the study participation and questionnaires on patients. Study participants will not be individually informed of the study results; however, dissemination to the public will be through media outreach (e.g., press release) upon publication of this study.

### Study population

The VESPA study recruited from the EPAU population. All women aged ≥16 years of age who attended the participating EPAUs presenting with first trimester pregnancy complications for the first time during the index pregnancy were included in the cohort study. The upper gestational age limit was set at 13^+6^ weeks’ gestation in order to keep recruitment from all participating EPAUs consistent. Non-identifiable demographic and routine clinical data were collected for all women attending the participating EPAUs, and if women gave their written consent to participate to the VESPA study, their clinical data were linked. Clinical follow-up was not standardised but was based on local management protocols and women’s medical needs.

For the emergency hospital care audit strand, data from routine hospital databases for women attending hospital emergency services (EDs) at the same location as the EPAU were collected in collaboration with the Information Services (IS) departments in the participating hospitals. We collected data about the total number of Emergency Department attendances and emergency in-patient admissions of women who presented with first trimester pregnancy complications. Wherever possible, all data relating to hospital admissions provided by the IS departments were crosschecked by a member of the research team against ward admission books/databases in an attempt to check their accuracy. The aim was to capture hospital emergency activity with regards to early pregnancy care in EDs. These data were collected retrospectively at the end of the three-month period corresponding to the date when the last woman recruited to the cohort study was discharged from each hospital’s EPAU.

### Emergency admission

The variable ‘Emergency admission’ indicates whether a patient had an unplanned admission to hospital following any visit for investigations or treatment of first trimester pregnancy complications. The primary outcome for the VESPA study was the number of emergency admissions as a proportion of women attending each participating EPAU. As a secondary outcome, we aimed to establish the number of emergency hospital admissions for women attending EDs in the same location as the EPAU for first trimester pregnancy complications.

### Senior clinician presence

Planned senior clinician presence was defined as the percentage of planned hours which they were expected to spend in the unit according to their weekly timetable, divided by the planned unit opening hours. This variable was also dichotomised for some analyses to indicate whether they were expected to spend any time in EPAU (yes/no). During patient recruitment, we collected data on the grade of all members of staff present in the EPAU during its opening hours, their involvement in delivering early pregnancy care, as well as the actual hours which each member of staff spent in the unit. We could therefore calculate the actual amount of time senior clinicians spent in EPAU delivering clinical care. Actual senior clinician presence was defined as the percentage of hours they spent in EPAUs delivering care divided by the unit opening hours.

### Other covariates

#### Unit volume

Unit volume was defined as the number of patient visits per year and was estimated using the time taken to obtain data on the required number of patients and the average number of visits per patient. This variable was also dichotomised for some analyses to indicate whether the annual number of patient visits for a unit was above or below the median.

#### Weekend opening

The total number of opening hours per weekend was used to illustrate weekend opening. For some analyses, this variable was also dichotomised to indicate whether a unit was open at weekends (yes/no), whilst a three-level weekend opening variable was defined: None/Saturday only/Saturday and Sunday.

#### Gestational age policy

At the time of patient recruitment, we collected information about the unit referral policy with regards to the minimum gestational age at which women could be seen at the EPAU. The variable Gestational Age Policy (GAP) captures whether units either accepted referrals for women of any gestational age or only reviewed women of six weeks’ gestation or more.

#### Final diagnosis

Final diagnosis (FD) was divided into five groups: 1) Normal intrauterine pregnancy including early and live pregnancies; 2) Abnormal intrauterine pregnancy including early embryonic demise, incomplete, and complete miscarriage; 3) Ectopic pregnancy at any site; 4) Pregnancy of unknown location; and 5) Other: molar pregnancy, multiple pregnancy, and not pregnant.

#### Index of Multiple Deprivation

Index of Multiple Deprivation (IMD) was calculated based on the home address postcode of the patient at the time of patient recruitment. IMD scores were divided into 10 decile groups.

### Statistical considerations

#### Sample size

In preparation for the study, we carried out a prospective audit of clinical activity in eight EPAUs in London, UK, which included 3,600 women. This showed substantial variations in the proportion of in-patient emergency admissions with 8.5% in units without, and 3.5% in those with, senior clinician presence. Based on this study, the sample size of 44 units, each contributing 150 patients, was calculated to give us 90% power to detect a 5% absolute difference in the risk of an emergency admission. A moderate level of clustering within units (Intraclass Correlation Coefficient, ICC = 0.04) was assumed.

#### Statistical analysis

The association between the three factors: senior clinician presence, unit volume, and weekend opening hours, and the proportion of emergency admissions was investigated by using regression models, where hierarchical models were used for analysing patient-level data, and standard models were used for analysing unit-level data. Most models were adjusted using one of two sets of potential confounder variables: i) maternal age and final diagnosis (MA+FD) or; ii) maternal age, final diagnosis, IMD and gestational age policy (MA+FD+IMD+GAP). Two sets were used because IMD and gestational age policy had a reasonable amount of missing data.

We performed sensitivity analyses by refitting the regression models using the binary variables for planned senior clinician presence, unit volume and weekend opening hours.

The relationship between emergency admissions from Emergency Departments and senior clinician presence, unit volume, and weekend opening hours was investigated by fitting multivariable logistic regression models. Models were either unadjusted or adjusted for gestational age unit policy.

### Ethics, funder, and role

The VESPA study received a favourable ethical opinion from the North West Research Ethics Committee (REC reference 16/NW/0587) in the UK, registration number ISRCTN 10728897. Where applicable, participants gave informed written consent before taking part in the study.

## Results

The study ran from December 2016 to July 2017. We obtained clinical data from 6,606 women. All units were asked to recruit a minimum number of 150 women each. Forty units met this target, whilst four units recruited between 143 and 149 women. 33 days (IQR 24–47) was the median time for units to complete recruitment.

Upon completion of data collection, we revisited the activity in all 44 units. The majority of units (37/44) had a unit volume of less than 4000 visits per year, and 22 units had a yearly visit volume lower than 2500 visits per year. In view of this, we revised our cut-off and used ≥2500 instead of ≥3000 visits to describe the units as high or low volume in the final data analysis. Twenty-three units were open during weekends. Senior clinicians were present only in 19 of the 44 EPAUs. This was due to the changes in provision of medical cover which occurred between the site initiation visit and the start of recruitment. A summary of all units showing their characteristics in respect to the key factors of interest and outlining participants’ median age, parity, deprivation score, ethnicity and gestational age at presentation is provided in Table 1. This is based on 6397 women that had complete information on the outcome and key characteristics.

**Table 1:** Summary of the units’ characteristics and median (inter-quartile range) age, ethnicity (%BAME), parity, deprivation decile and gestational age (N = 44).

Code	Senior Clinician Presence	Volume >2500	Weekend Opening	Women’s Age	Ethnicity (%BAME)	Parity	Deprivation Decile	Gestational Age
AIS	No	No	Yes	30 (26 to 35)	2.7	1 (0 to 1)	1 (1 to 2)	8 (6 to 10)
BDX	Yes	No	Yes	27 (23 to 33)	3.4	1 (0 to 2)	2 (2 to 5)	7 (6 to 9)
BVU	No	No	No	28 (24 to 32)	2.7	1 (0 to 1)	5 (3 to 7)	7.5 (6 to 9)
CXP	No	Yes	Yes	30 (26 to 35)	7.1	1 (0 to 1)	2 (1 to 2)	7 (6 to 9)
CZX	Yes	Yes	Yes	29 (25 to 34)	10.7	1 (0 to 2)	5 (2 to 8)	7 (6 to 10)
FVX	No	Yes	Yes	33 (29 to 37)	37.8	0 (0 to 1)	4 (2 to 5)	6 (5.5 to 8)
GFY	Yes	Yes	No	32 (28 to 36)	15.9	1 (0 to 1)	9 (7 to 10)	7 (6 to 9)
GLR	No	No	No	29 (24 to 33)	11.3	1 (0 to 2)	3 (1 to 5)	7 (6 to 9)
HJZ	No	No	Yes	29 (25 to 34)	57.0	1 (0 to 2)	2 (1 to 3)	8 (7 to 10)
HYG	No	No	No	31 (25 to 35)	11.3	1 (0 to 1)	8 (5 to 9)	7 (6 to 8)
IWX	No	No	No	31 (27 to 36)	32.0	1 (0 to 1)	8 (6 to 9)	6 (5 to 9)
JDG	No	Yes	Yes	29 (26 to 33)	26.0	1 (0 to 2)	3 (1 to 7)	7 (6 to 9)
JII	No	Yes	Yes	32 (29 to 35)	36.2	1 (0 to 1)	6 (4 to 7)	7 (6 to 8)
JNM	Yes	Yes	Yes	31 (26.5 to 35)	36.5	1 (0 to 2)	4 (3 to 7)	7 (6 to 9)
JPM	Yes	Yes	Yes	30 (26 to 35)	6.7	1 (0 to 2)	6 (4 to 8)	8 (7 to 10)
MJL	Yes	No	Yes	26 (22 to 31)	7.3	1 (0 to 1)	2 (1 to 5)	8 (7 to 9)
NSK	No	No	No	28 (23 to 33)	1.3	1 (0 to 2)	2 (2 to 4)	8 (6 to 10)
OVA	Yes	Yes	No	32.5 (28 to 37)	41.3	0 (0 to 1)	3 (2 to 6)	7 (6 to 9)
OYN	Yes	No	No	31 (27 to 35)	10.7	1 (0 to 2)	5 (3 to 7)	7 (6 to 8)
PCO	Yes	No	No	30 (26 to 34)	18.8	1 (0 to 2)	4 (1 to 7)	7 (6 to 9)
QAR	No	No	Yes	29 (25 to 34)	9.6	1 (0 to 2)	4 (2 to 6)	7 (6 to 9)
QSL	Yes	Yes	No	29 (25 to 34)	21.2	0 (0 to 1)	5 (3 to 8)	7 (6 to 10)
RPR	No	Yes	Yes	30 (26 to 35)	49.0	1 (0 to 2)	1.5 (1 to 4)	7 (6 to 9)
RXO	Yes	No	No	32 (28 to 36)	8.3	1 (0 to 1)	8 (7 to 10)	8 (7 to 9)
SCC	Yes	No	No	31 (27 to 35)	10.3	1 (0 to 1)	7 (4 to 9)	8 (6 to 9)
SDD	Yes	Yes	No	31 (27 to 35)	73.8	1 (0 to 1)	4 (3 to 6)	7 (6 to 9)
SHS	Yes	No	No	31 (26 to 35)	48.7	1 (0 to 1)	5 (3 to 6.5)	7 (6 to 9)
SXB	No	No	Yes	28 (25 to 33)	8.6	1 (0 to 2)	4 (3 to 8)	7 (6 to 9)
SXM	No	No	No	31 (25 to 35)	6.3	0 (0 to 1)	7 (6 to 9)	7 (6 to 9)
TCS	No	Yes	Yes	29 (26 to 34)	24.8	1 (0 to 2)	3 (2 to 7)	7 (6 to 8.5)
ULV	Yes	Yes	Yes	32 (28 to 36)	61.3	1 (0 to 2)	3 (2 to 3)	7 (6 to 8)
UOY	No	Yes	Yes	29 (25 to 34)	13.3	1 (0 to 2)	5 (3 to 8)	8 (6 to 10)
VXL	Yes	Yes	Yes	29 (25 to 34)	65.1	0 (0 to 2)	3 (2 to 3)	7 (6 to 9)
WDI	Yes	Yes	Yes	28 (24 to 33)	27.2	1 (0 to 1)	2 (1 to 4)	8 (6 to 9)
WWR	Yes	No	Yes	28 (24 to 33)	2.8	1 (0 to 2)	3 (1 to 5)	8 (6 to 10)
WYW	No	No	No	31 (28 to 36)	10.9	1 (0 to 1)	8 (6 to 10)	8 (6 to 9)
XNL	Yes	Yes	Yes	31 (26 to 34)	12.9	0 (0 to 1)	7 (6 to 9)	7 (6 to 9)
XQD	No	Yes	Yes	29 (25 to 35)	34.1	1 (0 to 2)	2 (1 to 4.5)	7 (6 to 10)
XQZ	No	Yes	Yes	32 (28 to 36)	1.3	1 (0 to 1)	7 (6 to 9)	8 (6 to 9)
YLJ	No	Yes	No	29 (24 to 34)	7.6	1 (0 to 2)	6 (3 to 8)	7 (6 to 9)
YUS	No	No	No	28 (23 to 31)	3.4	1 (0 to 2)	4 (2 to 5)	7 (6 to 9)
ZAM	No	No	No	28 (25 to 32)	3.5	1 (0 to 2)	5 (4 to 6)	7 (6 to 9)
ZAR	No	No	No	29 (25 to 32)	2.6	1 (0 to 2)	1 (1 to 1)	7 (6 to 8)
ZNI	No	Yes	No	31 (26 to 35)	4.1	1 (0 to 1)	5.5 (4 to 8)	7 (6 to 9)

**Fig 1:**
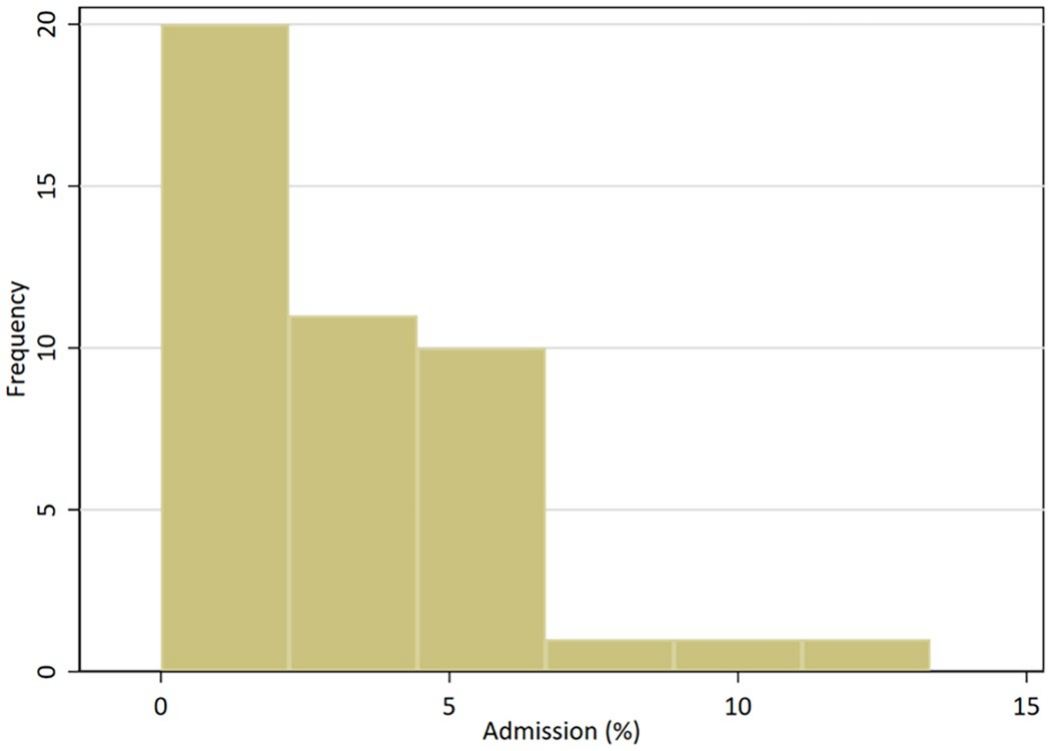
Emergency admission rates across 44 participating EPAUs.

A total of 205/6397 (3.2%; 95% CI 2.8–3.7) women were admitted following their EPAU attendance. The median admission rate among 44 units was 2.8% (range 0% to 13.7%) (Fig 1). The admission rates by patient characteristics is shown in Table 2. These are further broken down by diagnosis in Tables 3 and 4.

**Table 2:** Admission rates by patient characteristic (p-values from univariate tests; either Mann-Whitney or chi-squared tests).

Admission		No (n = 6192)	Yes (n = 205)	p-value
Senior Clinical Presence	No	3543 (97.1%)	107 (2.9%)	0.153
	Yes	2649 (96.4%)	98 (3.6%)	
Volume>25000	No	3113 (96.9%)	99 (3.1%)	0.577
	Yes	3079 (96.7%)	106 (3.3%)	
Weekend Opening	No	3012 (97.1%)	90 (2.9%)	0.181
	Yes	3180 (96.5%)	115 (3.5%)	
Women’s Age		30 (26 to 35)	30 (26 to 35)	0.627
BAME	No	4938 (96.9%)	156 (3.1%)	0.202
	Yes	1254 (96.2%)	49 (3.8%)	
Parity		1 (0 to 2)	1 (0 to 1)	0.516
Deprivation decile		4 (2 to 7)	4 (2 to 7)	0.782
Gestational Age (weeks)		7 (5 to 9)	6 (0 to 8)	0.069
Final Diagnosis	Normal	4157 (99.0%)	44 (1.0%)	<0.001
	Miscarriage	1840 (96.2%)	73 (3.8%)	
	Ectopic	39 (35.8%)	70 (64.2%)	
	PUL	131 (90.3%)	14 (9.7%)	
	Other	25 (86.2%)	4 (13.8%)	

**Table 3:** Patient characteristics of women diagnosed with normally-developing pregnancies (FD group 1).

Admission		No (n = 4157)	Yes (n = 44)
Senior Clinical Presence	No	2388 (99.0%)	24 (1.0%)
	Yes	1769 (98.9%)	20 (1.1%)
Volume>25000	No	2132 (99.1%)	20 (0.9%)
	Yes	2025 (98.8%)	24 (1.2%)
Weekend Opening	No	2044 (99.2%)	17 (0.8%)
	Yes	2113 (98.7%)	27 (1.3%)
Women’s Age		29 (25 to 34)	29 (25 to 34)
BAME	No	3324 (99.0%)	33 (1.0%)
	Yes	833 (98.7%)	11 (1.3%)
Parity		1 (0 to 2)	1 (0 to 2)
Deprivation decile		4 (2 to 7)	5 (2 to 8)
Gestational Age (weeks)		7 (5 to 9)	7 (6 to 10)

**Table 4:** Patient characteristics of women diagnosed with abnormally-developing pregnancies (FD groups 2–5).

Admission		No (n = 2035)	Yes (n = 161)
Senior Clinical Presence	No	1155 (93.3%)	83 (6.7%)
	Yes	880 (91.9%)	78 (8.1%)
Volume>25000	No	981 (92.5%)	79 (7.5%)
	Yes	1054 (92.8%)	82 (7.2%)
Weekend Opening	No	968 (93.0%)	73 (7.0%)
	Yes	1067 (92.4%)	88 (7.6%)
Women’s Age		32 (26 to 36)	30 (26 to 35)
BAME	No	1614 (92.9%)	123 (7.1%)
	Yes	421 (91.7%)	38 (8.3%)
Parity		1 (0 to 2)	1 (0 to 1)
Deprivation decile		5 (2 to 8)	4 (2 to 7)
Gestational Age (weeks)		6 (0 to 8)	6 (0 to 8)

**Fig 2:**
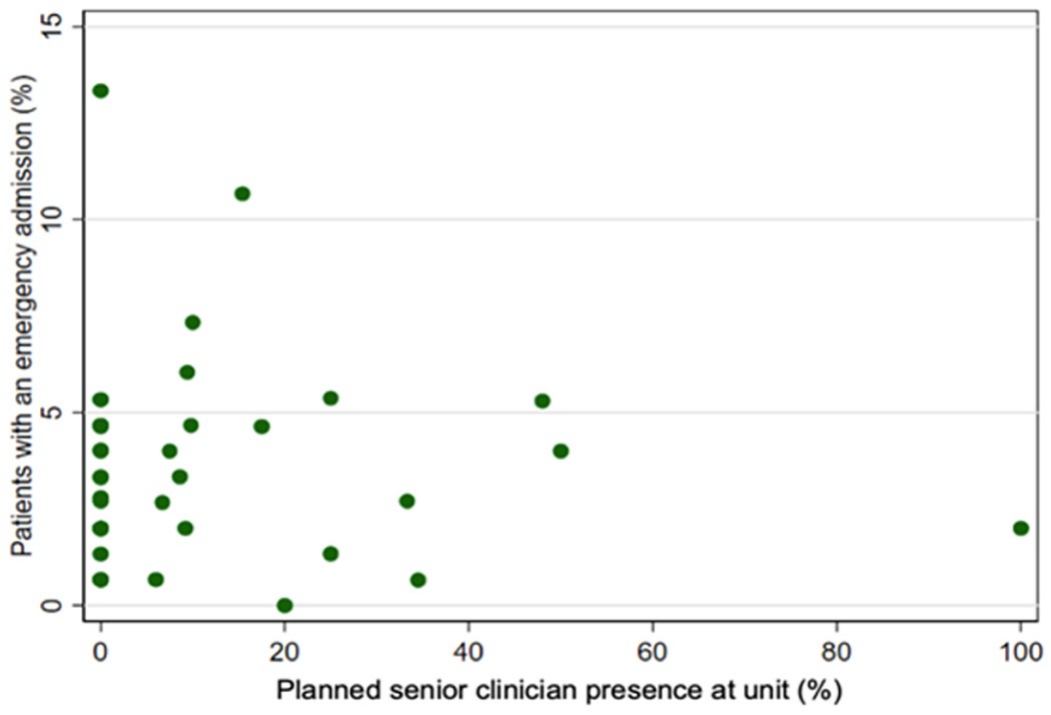
Emergency admissions from EPAUs vs planned senior clinician presence for each unit.

Initially, we investigated the association between senior clinician presence, unit volume, and weekend opening hours on emergency admissions from EPAU. There was no apparent relationship between planned senior clinician presence and emergency admissions (Fig 2).

Fitting hierarchical logistic regression models for ‘Emergency admission from EPAU’, there was little evidence of an association between the admission rate and planned senior clinician presence or unit volume controlled for final diagnosis and maternal age, as shown in Table 5. However, there was evidence of an association between the emergency admission rate and weekend opening hours. That is, a one-hour increase in weekend opening hours was associated with a 3.0% (95% CI: 0.3% to 5.8%) increase in the odds of an emergency admission from EPAU. These associations did not markedly change after further confounder adjustment. They were also similar if we consider patients with normally-developing pregnancies (FD group 1) and abnormally-developing pregnancies (FD groups 2–5) (Table 6).

**Table 5:** Odds ratios from multivariable hierarchical logistic regression models for emergency admission from EPAU.

Variable	OR (95%CI)	p-value
Planned senior clinician presence (%)	1.001 (0.986 to 1.017)	0.874
Volume (per 100 visits)	0.990 (0.974 to 1.007)	0.247
Weekend opening (hours)	1.030 (1.003 to 1.058)	0.027

**Table 6:** Odds ratios from multivariable hierarchical logistic regression models for emergency admission from EPAU for women diagnosed with normally-developing (FD group 1) or abnormally-developing pregnancies (FD groups 2–5) (planned senior clinician time: p = 0.63, volume: p = 0.11, weekend opening: p = 0.62).

Variable	OR (95% CI)	p-value
**Normally-developing pregnancies (n = 4201)**		
Planned senior clinician time (%)	0.999 (0.971 to 1.028)	0.950
Volume (per 100 visits)	1.000 (0.974 to 1.028)	0.973
Weekend opening (hours)	1.023 (0.978 to 1.071)	0.324
**Abnormally-developing pregnancies (n = 2196)**		
Planned senior clinician time (%)	1.002 (0.988 to 1.016)	0.778
Yearly volume (/100)	0.987 (0.972 to 1.003)	0.103
Weekend opening (hours)	1.030 (1.006 to 1.055)	0.014

**Fig 3:**
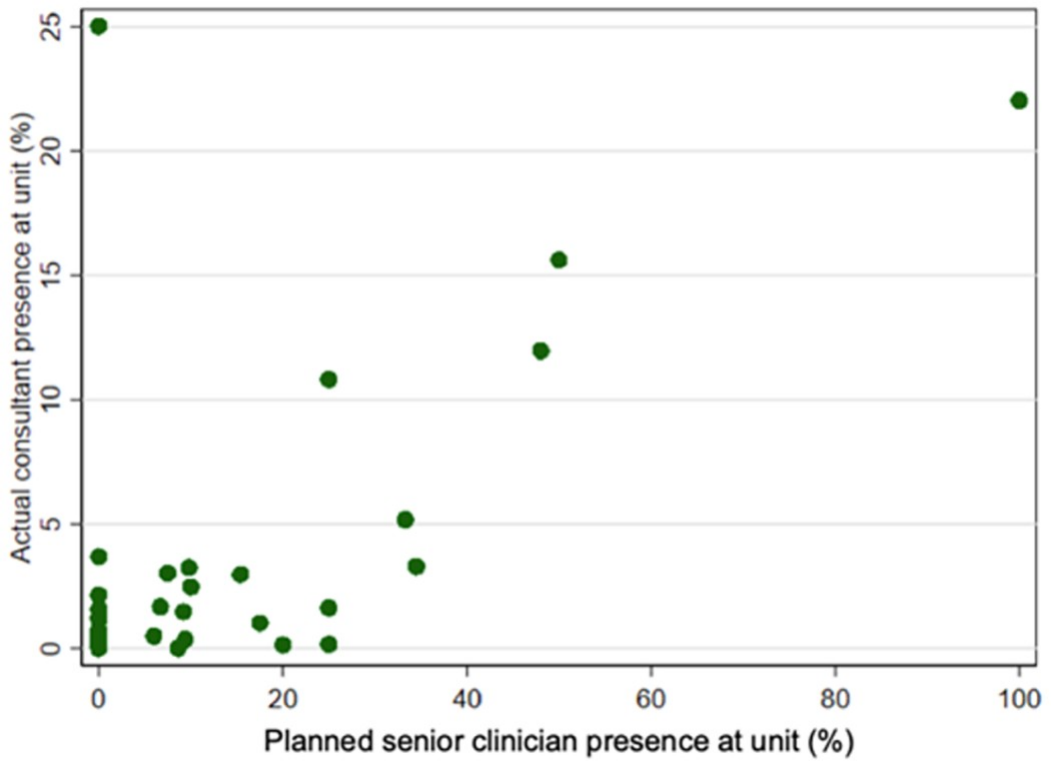
Actual vs planned senior clinician presence in participating EPAUs.

We found that, in general, the actual amount of time spent by the senior clinicians in the units was less than planned, and in only five units, they were present for more than 5% of the time (Fig 3).

**Fig 4:**
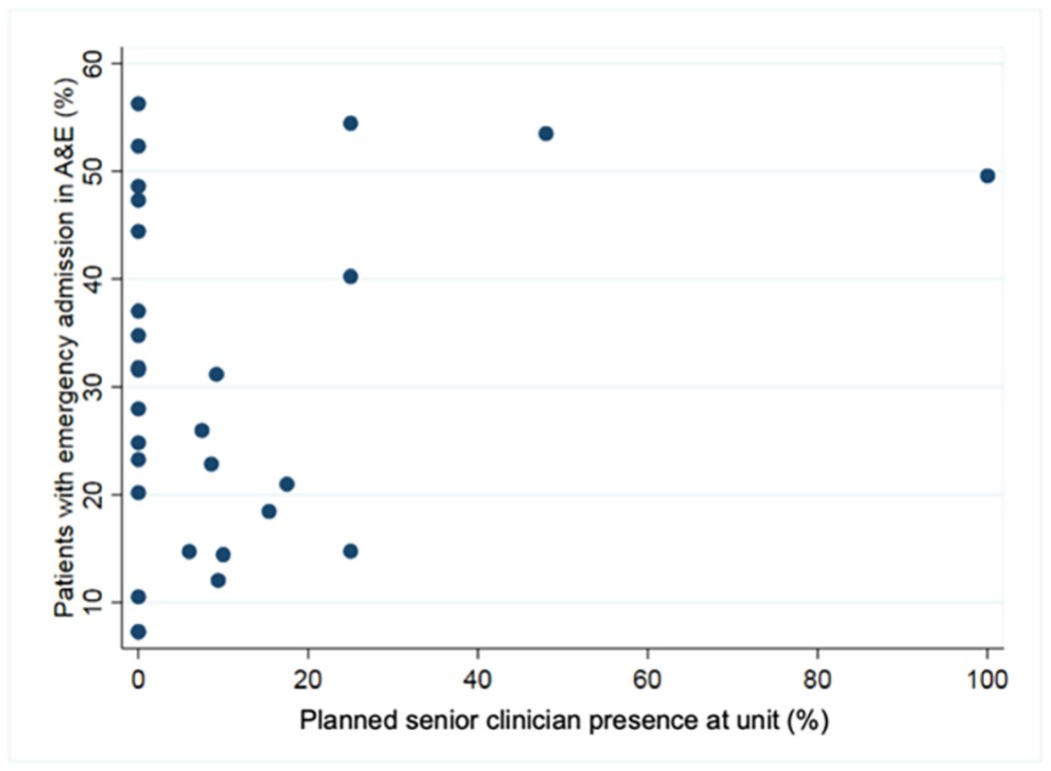
Emergency admission from ED vs senior clinician presence for each unit.

We next investigated the relationship between emergency admissions from the Emergency Department and planned senior clinician presence in EPAU, unit volume and weekend opening hours of the EPAU. This analysis was based on the 29 units (5,464 patients) that were able to provide us with reliable data regarding the attendances to Emergency Department and emergency admissions out of hours. In total, 1,445/5,464 (26.4%; 95%CI 25.3 to 27.6) patients had an emergency admission from the Emergency Department. The percentage of emergency admissions from the Emergency Department ranged from 7% to 58% with the majority of the units having an emergency admission rate of between 10% and 30%. The relationship between emergency admissions from the Emergency Department and senior clinicians’ presence in EPAU is shown in Fig 4.

Fitting multivariable logistic models, we found an association between the emergency admissions from the Emergency Department and weekend opening (p = 0.037). A 1-hour increase in the weekend opening hours of the EPAU was associated with 2.4% (95% CI: 0.1% to 4.7%) lower odds of an emergency admission from the Emergency Department. However, there was little evidence of an association with unit volume (p = 0.647) or planned senior clinician time (p = 0.280).

## Discussion

We found no significant association between senior clinician presence in EPAUs and the proportion of women admitted for suspected first trimester pregnancy complications. We obtained similar results whether we analysed for senior clinician presence as a binary outcome or as a continuum. Having collected contemporaneous data documenting the actual time that senior clinicians spent in the EPAU delivering early pregnancy care, we found no significant association whether we analysed for planned or actual presence of the senior clinicians in EPAU. Statistical models were adjusted for final diagnosis, maternal age at initial visit, deprivation score, and unit referral policy regarding gestational age, as these variables have a known effect on the rate of emergency admissions [15–17].

Our findings are in contrast with the results of previous studies in other clinical settings, which showed that involvement of more experienced clinicians in delivering emergency care results in a significant reduction in emergency hospital admissions and the length of stay in hospital [12, 18].

However, our study is the first large prospective study designed with a specific aim to study emergency admission rates in women presenting with acute first trimester pregnancy complications. We chose to study the rate of emergency admissions which is a recognised quality outcome indicator for acute hospital units [19, 20]. In addition, we recorded the amount of time senior clinicians (consultant gynaecologists) spent working in EPAUs which is of critical importance when assessing the impact of their presence on various clinical outcomes.

In addition to senior clinicians’ presence, we also explored the effect that weekend opening of an EPAU has on emergency admissions. Having collected detailed data on the opening hours of all participating EPAUs over the weekend, we were able to identify that one hour’s increase in the weekend opening hours of an EPAU resulted, as expected, in an increased probability of a woman with first trimester complications being admitted to hospital from the EPAU, but decreased the chance of an admission to hospital from the Emergency Department. Bearing in mind that women attending the ED are eight times more likely to be admitted than those seen in the EPAU, keeping EPAUs open over the weekend reduces the overall number of emergency admissions to hospital.

Our study has several limitations. The senior clinicians were actually spending less time in the EPAUs than expected. In view of that, we were unable to identify the threshold above which senior clinicians would need to be present in the units actively delivering early pregnancy care in order to improve clinical outcomes. In addition, obtaining good quality data on emergency admissions from the Emergency Department based on routine hospital databases proved to be challenging, and we were only able to confidently utilise the data obtained from a fraction of the participating units.

There are several possible explanations for our findings. When we compared actual with planned senior clinicians’ presence, we found that in only 3/19 units (16%) did senior clinicians spend more than 15% of their time actively delivering clinical care, and in 13/19 units (68%) their actual documented presence was less than 5%. This relatively low level of senior clinicians’ involvement could explain the lack of an observed association between their presence and emergency admissions in this study. The other possibility is that senior clinicians, who are fully trained in general obstetrics and gynaecology, lack the additional clinical and ultrasound skills necessary for the effective running of an EPAU. Despite the fact that an advanced training module in acute gynaecology and early pregnancy has been running for a number of years from The Royal College of Obstetricians and Gynaecologists, to our knowledge, a rigorous assessment of its quality and effectiveness has not been undertaken [21]. It is also unclear whether completion of the module is a requirement locally for appointment to EPAU leadership roles.

There is also a need to further explore the skill mix of a multidisciplinary team of nurses, midwives and sonographers, which could facilitate effective running of EPAUs in the absence of senior clinicians.

The results of our study indicate that the presence of senior clinicians in early pregnancy units may not lead to more effective care when this is measured by the proportion of women who are admitted as an emergency. This is an important finding for health providers as the cost of employing senior clinicians is much higher compared to nurses and other healthcare professionals. Equally, weekend opening of an EPAU, even for a limited number of hours, is associated with lower probability of emergency hospital admissions.

Future research is needed to assess whether increased participation of senior clinicians and actual time commitment in provision of early pregnancy care, a change in their training could have more significant impact on the rate of emergency admissions and other indicators of the quality of clinical care in early pregnancy.

## Supporting information

S1 Dataset (XLSX)

S2 Dataset (XLSX)
